# Pre-infection liver function is associated with all-cause mortality among hemodialysis patients with SARS-CoV-2 Omicron variant infection

**DOI:** 10.1080/0886022X.2024.2425069

**Published:** 2024-11-18

**Authors:** Quanchao Zhang, Caibao Lu, Han Wang, Shaofa Wu, Lili Jiang, Jie Li, Zhifen Wu, Bingshuang Tang, Bingfeng Yang, Shengli Liao, Liao Wang, Hongwei Chen, Moqi Li, Wenchang He, Yiqin Wang, Jin He, Jinghong Zhao, Ling Nie

**Affiliations:** a Department of Nephrology, the Key Laboratory for the Prevention and Treatment of Chronic Kidney Disease of Chongqing, Chongqing Clinical Research Center of Kidney and Urology Diseases, Xinqiao Hospital, Army Medical University (Third Military Medical University), Chongqing, China; b Department of Endocrinology and Nephrology, Chongqing General Hospital, Chongqing, China; c Department of Nephrology, Youyang Hospital, A Branch of the First Affiliated Hospital of Chongqing Medical University, Chongqing, China; d Urology and Kidney Disease Center, Yongchuan People’s Hospital of Chongqing, Chongqing, China; e Department of Nephrology and Endocrinology, ChongQingBishan District Hospital of Traditional Chinese Medicine, Chongqing, China; f Hemodialysis Center of Nanchuan Hospital of Traditional Chinese Medicine, Chongqing, China

**Keywords:** Hemodialysis, Omicron, liver function, mortality

## Abstract

**Background:**

There is ample evidence to suggest that patients infected with SARS-CoV-2 Omicron variant may experience liver dysfunction. However, the impact of pre-infection liver function on postinfection mortality rates remains inadequately researched.

**Methods:**

Data from 847 hemodialysis (HD) patients, diagnosed with Omicron across six HD centers between December 2022 and February 2023, were analyzed. Initial liver function assessments were conducted, following which patients were monitored for mortality outcomes. The stepwise multivariable Cox regression analysis and receiver operating characteristic (ROC) curves were utilized to identify the predictors of mortality.

**Results:**

From the total, 98 patients (11.6%) succumbed, with a majority (80/98) within a month postinfection. The deceased patients were observed to be mostly older males with an increased prevalence of diabetes and tumors, signifying higher AST and C-reactive protein levels. These patients also exhibited lower hemoglobin, albumin, and prealbumin levels. An elevated AST [per 1 IU increment; HR 1.04 (95% CI 1–1.04), *p* = 0.026], AST/ALT ratio [per 1 increment; HR 1.52 (95% CI 1.27–2.36), *p* = 0.004], and reduced prealbumin [per 10 mg/L increment; HR 0.93 (95% CI 0.9–0.96), *p* < 0.001] were discovered to be independent indicators of an increased mortality risk. Notably, AST, AST/ALT ratio, and prealbumin proved significant predictors of mortality (AUC values were 0.59, 0.65, and 0.79 respectively).

**Conclusions:**

This study underscores that pre-infection liver function, specifically AST, AST/ALT ratio, and prealbumin levels, substantially influence the mortality rates in HD patients following Omicron infection. Therefore, careful consideration of these liver function parameters could guide superior patient management strategies and potentially decrease mortality rates within this at-risk population.

## Introduction

The COVID-19 pandemic, sparked by the SARS-CoV-2 virus, has greatly affected both global health and societal norms [1]. It has resulted in millions of infections and caused widespread disruption globally. As of February 11, 2024, the World Health Organization has documented over 774 million confirmed cases of coronavirus disease 2019 (COVID-19), resulting in more than 7 million COVID-19-related deaths [2].

During the COVID‐19 epidemic, several SARS-CoV-2 variants emerged, including Omicron (B.1.1.529). It was first detected in specimens on November 11, 2021, in Botswana and on November 14, 2021, in South Africa [3]. The WHO defined it as the fifth variant of concern and named it Omicron. On December 7, 2022, China’s COVID Zero strategy was relaxed, and the epidemic rapidly spread throughout the country. At this time, the Omicron variant had become the main strain during the global pandemic, and the same was true in China.

SARS-CoV-2 affects not only the lungs but also other organs, potentially leading to multi-organ failure and increased mortality [4,5]. Among them, liver impairments in COVID-19 patients are continuously documented. The earliest available epidemiological data of COVID-19 patients came from China. Abnormal liver function was first reported in a cohort from Wuhan, China [6], making liver the most frequently damaged outside of the respiratory system. With respect to worldwide published data the overall prevalence of abnormal liver function tests ranges from 2.5% to 96.8%. For omicron, the incidence rate is about 11–15% [7,8].

However, there is currently no research indicating whether pre infection liver function affects post infection outcomes, especially in hemodialysis (HD) patients. They often suffer from multiple comorbidities and are of advanced age, their vulnerability to infection is heightened, resulting in higher hospitalization rates and increased morbidity and mortality due to weakened immunity caused by chronic kidney disease (CKD) [9,10].

With this in mind, the main aim of this study is to explore the relationship between pre-infection liver function and postinfection outcomes in HD patients infected with the Omicron variant. The potential outcomes of this research could fill gaps in the existing knowledge, thus enriching our understanding of how the Omicron variant influences liver health in HD patients.

## Materials and methods

**Figure 1.**
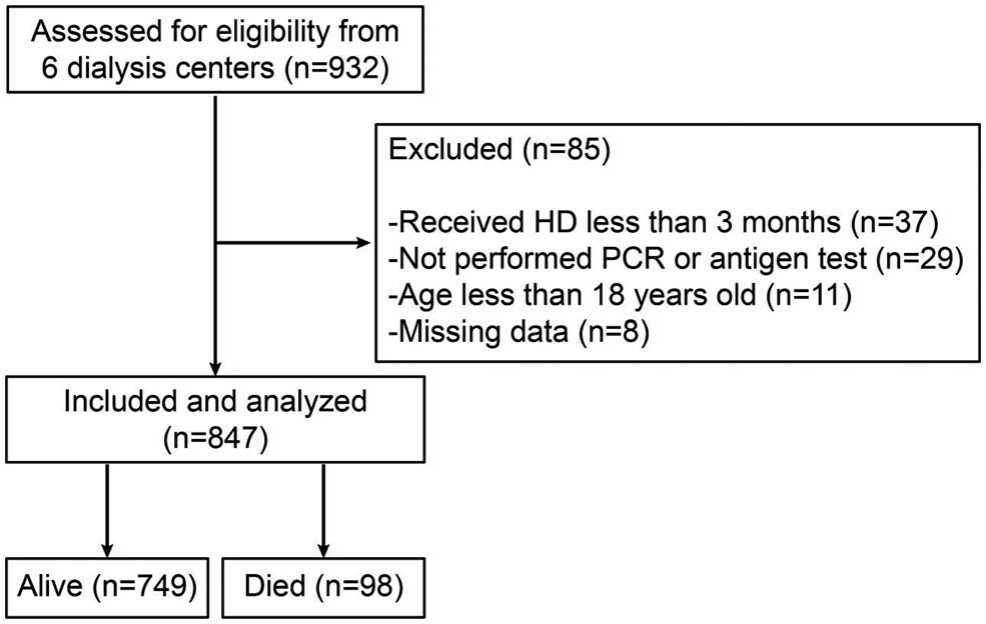
The flowchart of inclusion and exclusion of participants.

The methodology used in this study has been described in our previous research [11]. Simply, the team performed a retrospective analysis of a cohort of Omicron infected HD patients aged above 18 years old, received HD for at least 3 months and confirmed SARS-CoV-2 infection for the first time. Those below 18, uninfected by Omicron or has been infected before the start of the study, or without sufficient clinical information were excluded. The inclusion and exclusion process is shown in Figure 1. Demographic, clinical, laboratory information, and comorbidities were collected. This included age, gender, BMI, dialysis information, kidney disease etiology, and a list of comorbidities. Various drugs used by the patients were also noted. The primary exposure was the first-time infection by Omicron, and the primary outcomes were hospitalization due to COVID-19 and all-cause mortality following infection. Patients were divided into different subgroups according to: age greater or less than 65 years, sex, the presence or absence of comorbidities including diabetes, cardio-cerebrovascular disease (CCVD), chronic obstructive pulmonary disease (COPD) or tumor, and whether vaccinated against SARS-CoV-2 or not. Continuous variables are represented as the average ± SD or the median and IQR depending on the normal distribution results using the Kolmogorov–Smirnov test, whereas categorical variables are described as percent ages (%). The t-test was applied for normally distributed data, and the Mann-Whitney U test was applied for non-normally distributed data. The χ^2^ test was applied for categorical variable data. Hazards ratios were estimated by multivariable Cox regression, and receiver operating characteristic curves were used to evaluate predictive abilities of the models. The analysis method in subgroup was the same as that for the whole population. Data was analyzed statistically using SPSS Statistics 23 (IBM Company, Chicago, USA), and significance was placed at *p* < 0.05.

## Results

During the period from December 2022 to February 2023, we included a total of 847 HD patients with confirmed Omicron infection from 6 HD centers in the study. The median age of the participants was 57 years old, and 58% of the participants were male. The median dialysis vintage was 50 months. The majority (89%) of participants underwent thrice-weekly dialysis sessions, and 96.9% had an autologous arteriovenous fistula or arteriovenous graft for permanent vascular access. In terms of chronic kidney disease (CKD) etiology, glomerulonephritis was present in 268 patients (31.6%), diabetic nephropathy in 152 patients (17.9%), hypertensive nephropathy in 101 patients (11.9%), while 219 patients (38.5%) had other or unknown causes.

Throughout the follow-up period, 98 (11.6%) patients died. The vast majority (80/98) of patients died within one month. Compared with the alive patients, those died were older, higher proportion of males, more patients had diabetes, tumor as well as higher levels of AST, AST/ALT ratio and C-Reactive protein. Additionally, the levels of hemoglobin, albumin and prealbumin were lower in these individuals. Lastly, these individuals had a higher usage of inhalation drugs and fewer vaccinations against Omicron. Table 1 provides a summary of the patients’ characteristics and laboratory data.

**Table 1.** The general clinical characteristics of the included participants.

	Total (*n* = 847)	Alive (*n* = 749)	Dead (*n* = 98)	*p*
Age (year)	57 (48, 67.5)	55 (47, 65)	69.5 (59.2, 75.8)	**<0.001**
Male, %	491 (58%)	423 (56.5%)	68 (69.4%)	**0.015**
BMI (kg/m^2^)	22.3 (20, 24.3)	22.3 (20, 24.3)	22.3 (20.2, 24.1)	0.477
Dialysis vintage (months)	50 (24, 96)	51 (24, 96)	48 (27.8, 83.2)	0.353
Vascular access				1
AVF/AVG	821 (96.9%)	726 (96.9%)	95 (96.9%)	
Catheter	26 (3.1%)	23 (3.1%)	3 (3.1%)	
Dialysis frequency				0.379
3/week	754 (89%)	663 (88.5%)	91 (92.9%)	
5/2 weeks	9 (1.1%)	9 (1.2%)	0 (0%)	
2/week	73 (8.6%)	66 (8.8%)	7 (7.1%)	
1/week	11 (1.3%)	11 (1.5%)	0 (0%)	
Combination with PD	22 (2.6%)	21 (2.8%)	1 (1%)	0.482
CKD etiology				**0.011**
Glomerulonephritis	268 (31.6%)	244 (32.6%)	24 (24.5%)	
Diabetes	152 (17.9%)	127 (17%)	25 (25.5%)	
Hypertension	101 (11.9%)	90 (12%)	11 (11.2%)	
Other	107 (12.6%)	87 (11.6%)	20 (20.4%)	
Unknown	219 (25.9%)	201 (26.8%)	18 (18.4%)	
Comorbidities				
Diabetes	147 (17.4%)	116 (15.5%)	31 (31.6%)	**<0.001**
CCVD	39 (9.2%)	34 (8.8%)	5 (13.2%)	0.558
COPD	22 (2.6%)	17 (2.3%)	5 (5.1%)	0.187
Tumor	11 (1.3%)	6 (0.8%)	5 (5.1%)	**0.002**
Laboratory data				
Hemoglobin, g/L	110 (97, 121)	111 (98, 121)	105.5 (91.8, 117.5)	**0.029**
Platelet, *10^9^/L	160 (127, 210.8)	160 (127, 210)	160.5 (127.2, 218.2)	0.938
Ca, mmol/L	2.2 (2.1, 2.3)	2.2 (2.1, 2.3)	2.2 (2, 2.3)	0.164
P, mmol/L	1.7 (1.4, 2.1)	1.7 (1.4, 2.1)	1.7 (1.4, 2)	0.457
iPTH, pg/ml	382.1 (168, 638.7)	386.6 (175.8, 649.1)	242.6 (154.8, 517.7)	0.106
Kt/V_urea_	1.3 (1.2, 1.5)	1.3 (1.2, 1.5)	1.3 (1.2, 1.4)	0.254
ALT, U/L	12 (8.6, 18)	12 (8.7, 18)	11.4 (7.7, 18.6)	0.434
AST, U/L	13.4 (10, 18)	13.2 (10, 18)	15.5 (10.6, 24.4)	**0.028**
AST/ALT ratio	1.1 (0.8, 1.4)	1.1 (0.8, 1.4)	1.3 (1.2, 1.4)	**<0.001**
Albumin, g/L	38.2 (35.5, 40.9)	38.8 (36.3, 41.2)	34.1 (30.1, 37.6)	**<0.001**
C-Reactive Protein, g/L	6.3 (2.8, 18.2)	6 (2.7, 16)	28.8 (14.7, 74.6)	**<0.001**
Prealbumin, mg/L	284.8 (230, 345)	297.7 (246.4, 352.6)	214.1 (173.8, 249.2)	**<0.001**
Ferritin, g/L	88 (39, 213.6)	87.2 (39, 213.5)	100 (42.5, 221.4)	0.592
Drugs use				
ACEI/ARB	154 (18.2%)	142 (19.0%)	12 (12.2%)	0.965
Inhalable drugs	20 (2.4%)	15 (2.0%)	5 (5.1%)	**0.014**
Immunosuppressant	8 (0.9%)	8 (1.1%)	0 (0%)	0.177
Glucocorticoids	20 (2.4%)	18 (2.4%)	2 (2.0%)	0.561
Antiviral drugs	18 (2.1%)	14 (1.9%)	4 (4.1%)	0.088
Vaccinated against SARS-CoV-2	196 (23.1%)	191 (25.5%)	5 (5.1%)	**<0.001**

BMI: body mass index; AVF/AVG: autologous arteriovenous fistula/arteriovenous graft; PD: peritoneal dialysis; CKD: chronic kidney disease; CCVD: cardio-cerebrovascular disease; COPD: chronic obstructive pulmonary disease; Ca: calcium; P: phosphorous; iPTH: intact parathyroid hormone; ALT: alanine transaminase; AST: aspartate transaminase; ACEI/ARB: angiotensin-converting enzyme inhibitors/angiotensin-receptor blockers. *P*-values less than 0.05 are bolded.

Above results had demonstrated a positive association (*p* < 0.05) between mortality and variables containing age, sex, CKD etiology, diabetes, tumor, AST, AST/ALT ratio, C-Reactive protein, hemoglobin, albumin, prealbumin, inhalation drugs, and vaccination. Subsequently, we performed a stepwise multivariable Cox regressive analysis. The results revealed that three liver function related parameters including AST [per 1 IU increment; HR 1.04 (95% CI 1-1.04), *p* = 0.026], AST/ALT ratio [per 1 increment; HR 1.52 (95% CI 1.27-2.36), *p* = 0.004] and prealbumin [per 10 mg/L increment; HR 0.93 (95% CI 0.9-0.96), *p* < 0.001] were independently associated with higher rates of mortality. These results are presented in Table 2.

**Table 2.** Multivariate analysis of the associations between variables and all-cause mortality using stepwise Cox regression analyses.

	HR [95% CI]	*p*
Age, per 1 year	1.07 [1.04, 1.1]	<0.001
Sex	2.26 [1.04, 4.89]	0.039
ALT, per 1 IU	1.81 [0.93, 3.52]	0.079
AST, per 1 IU	1.04 [1, 1.07]	0.026
AST/ALT ratio, per 1	1.52 [1.27, 2.36]	0.04
C-Reactive Protein, per 1 g/L	1.01 [1, 1.01]	0.001
Prealbium, per 10 mg/L	0.93 [0.9, 0.96]	<0.001
Vaccinated against SARS-CoV-2	0.33 [0.11, 0.94]	0.037

For covariates of sex and vaccinated against SARS-CoV-2, male and unvaccinated against SARS-CoV-2 were used as references, respectively.

**Figure 2.**
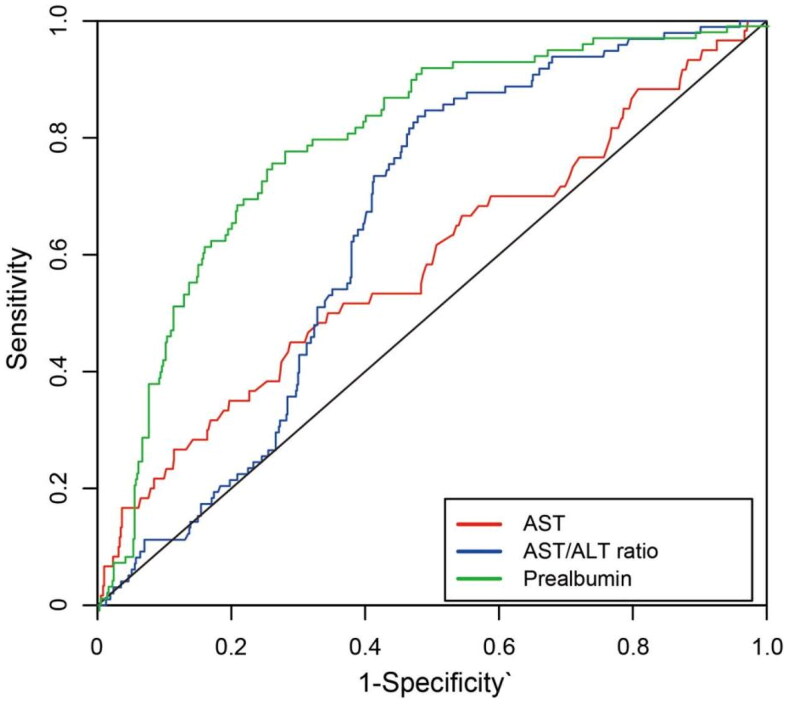
The predictive capability of AST, AST/ALT ratio and prealbumin for all-cause mortality demonstrated by ROC curves.

**Figure 3.**
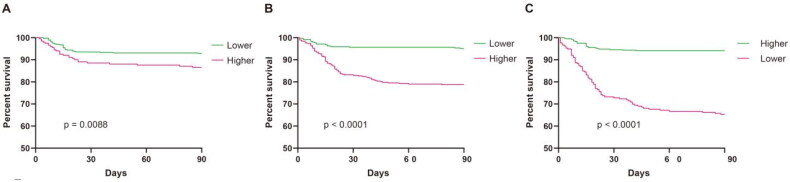
Comparison of survival rate between higher and lower of AST (a), AST/ALT ratio (B) or prealbumin (C).

Furthermore, we conducted an evaluation to assess the predictive capability of these three parameters for all-cause mortality using receiver operating characteristic (ROC) curves. The area under the curves (AUC) were 0.59 (95% CI 0.50–0.67), 0.65 (95% CI 0.60–0.70) and 0.79 (95% CI 0.75–0.84) for AST, AST/ALT ratio and prealbumin, respectively (Figure 2). The identified cutoff values for AST, AST/ALT ratio, and prealbumin were 16.9 IU, 1.11, and 249.5 mg/L, respectively. Moreover, we transformed these three parameters into categorical variables based on these cutoff values and divided the whole population into higher and lower groups. Using Kaplan-Meier curves, we compared all-cause mortality among the various categories defined by these three independent influencing factors. The results indicated a significant increase in all-cause mortality within the higher AST, higher AST/ALT ratio and lower prealbumin groups (Figure 3).

**Figure 4.**
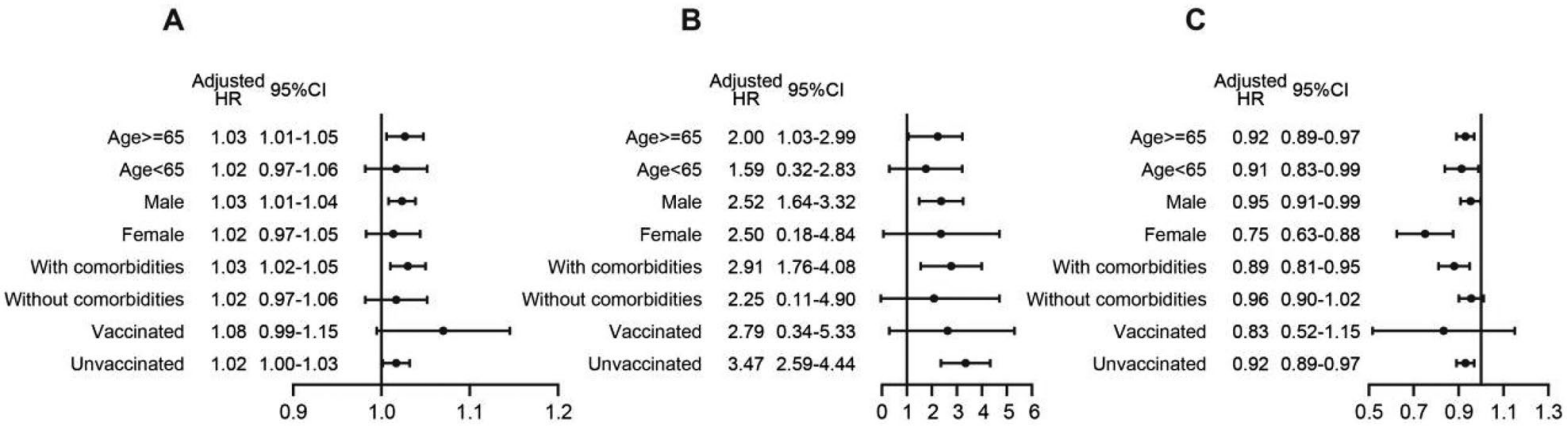
Effectiveness of AST (a), AST/ALT ratio (B) and prealbumin (C) in specific populations.

Lastly, we conducted subgroup analyses to determine the effectiveness of these three parameters in specific populations. Patients were grouped by age, sex, comorbidities and vaccination against SARS-CoV-2. For the elderly, male, with comorbidities and unvaccinated subgroups, higher AST, higher AST/ALT ratio and lower prealbumin were associated with higher all-cause mortality (Figure 4). These results demonstrated that liver function parameters may had better predictive power in specific populations than in the entire population.

## Discussion

In this study, three liver function parameters, namely AST, AST/ALT ratio, and prealbumin, were found to predict all-cause mortality following omicron infection in HD patients, even after adjusting for confounders. Moreover, these findings were held up in a subgroup analysis. To our knowledge, this is the first study to identify risk factors affecting the outcomes of HD patients preceding Omicron infections.

We noted a mortality rate of 11.6% (98/847). Further, 97.0% (80/98) of these deaths occurred within 30 days. *via* t-tests, we determined that there were significant differences in demographics and biochemical laboratory results between the deceased and surviving patient groups. The distinguishing factors were age, sex, comorbidities (including diabetes and tumor), and levels of AST, AST/ALT ratio, C-reactive protein, hemoglobin, albumin, and prealbumin. Adjusted Cox regression analyses revealed that AST, AST/ALT ratio, and prealbumin levels—but not ALT—may serve as prognostic markers for increased mortality among HD patients with COVID-19. These three factors can suggest the presence of liver dysfunction. Moreover, liver damage potentially resulting from the infection could further exacerbate liver dysfunction and ultimately elevate mortality risk. This finding aligns with previous studies that reported abnormal liver function in HD patients post-COVID-19 infection [12],

Since the onset of the SARS-CoV-2 outbreak, significant liver injury—as indicated by abnormal elevations of biomarker enzymes in patient sera—has been noted in many COVID-19 patients [13,14]. Hepatocellular, a type of liver damage, is indicated by increased levels of ALT and AST [15]. AST, present in numerous tissues, is both cytosolic (20%) and mitochondrial (80%) localized in the liver [16]. Although AST/ALT levels do not correspond with the histological parameters of liver disease severity, changes in this ratio can differentiate between cirrhotic and non-cirrhotic patients [17]. Previous studies have also found that AST/ALT levels effectively predict cirrhosis and that the AST/ALT ratio correlates with the histological grade of necroinflammatory activity and fibrosis [18]

The prognostic utility of prealbumin among HD patients warrants robust exploration. Serum prealbumin plays an integral role in the malnutrition assessment and dietary screening in routine clinical practice [19]. Physiological stress, liver dysfunctions, infections, and malnutrition may induce a decline in prealbumin levels [20]. Prealbumin’s diagnostic efficacy among patients with inflammation-driven maladies remains contentious, given that inflammatory conditions influence prealbumin levels. Conversely, prealbumin could mirror the overlapping effects of these two conditions common among HD patients, presenting a compelling case for its relevance in this cohort. Recent studies have positioned prealbumin as a promising prognostic tool for COVID-19 patients, demonstrating low prealbumin levels on ICU admission correspond with elevated mortality risk [21]. Furthermore, a meta-analysis involving 2104 patients across nine studies affirmed that lower serum prealbumin was correlated with adverse outcomes, characterized by a heightened mortality rate and disease progression among COVID-19 patients [22]. Thus, prealbumin holds considerable promise for predicting outcomes among COVID-19 patients, particularly those receiving HD.

Liver injury stemming from SARS-CoV-2 infection implicates multifaceted mechanisms. Principally, SARS-CoV-2 can directly interact with liver progenitor cells *via* ACE2, instigating cholangiocyte death through apoptosis mobilization and lysis, and cellular necrosis [23]. Secondarily, liver hypoxia may induce hepatocyte inflammation, lipid accumulation, augmented reactive oxygen species, and cell death [24,25]. Viral triggers can activate the immune defense system and oxidative stress response, culminating in the release of an array of cytokines [26]. Extreme hypercytokinemia, known as a cytokine storm, instigates a cascade of reactions leading to tissue damage (especially in the liver) and Multiple Organ Dysfunction Syndrome [27]. Besides, SARS-CoV-2 can provoke endothelial cell damage, altering microcirculation and fostering thrombus formation, organ ischemia, and tissue edema [28–30]. Additional liver damage may arise from oxidative stress [31,32], antiviral drugs usage [33], or modifications to the gut microbiome [34,35]

Several limitations of the current study are worth noting. The observational nature of the study could introduce unavoidable biases affecting the validity of the conclusions drawn. Moreover, this study did not examine the reported liver injury manifestations post-COVID-19 infection, such as abnormal levels of alkaline phosphatase and γ-glutamyl transferase.

In conclusion, our study uncovers a discernable association between pre-infection liver function and overall mortality among Omicron-infected HD patients. The findings shed light on risk stratification and potential intervention strategies for this high-risk population, underscoring the need for rigorous monitoring and management endeavors.

## Data Availability

All data generated or analyzed during this study are included in this published article.
